# Effect of attention-deficit–hyperactivity-disorder training program on the knowledge and attitudes of primary school teachers in Kaduna, North West Nigeria

**DOI:** 10.1186/s13034-017-0153-8

**Published:** 2017-03-20

**Authors:** Dupe Lasisi, Cornelius Ani, Victor Lasebikan, Lateef Sheikh, Olayinka Omigbodun

**Affiliations:** 1 Federal Neuropsychiatric Hospital, Barnawa, Kaduna Nigeria; 2 Academic Unit of Child and Adolescent Psychiatry, Imperial College London, London, UK; 3 Department of Psychiatry, University of Ibadan and University College Hospital, Ibadan, Nigeria; 4 Centre for Child and Adolescent Mental Health, University of Ibadan, College of Medicine, University College Hospital, Ibadan, Nigeria

**Keywords:** ADHD, Training, Teachers, Kaduna, Nigeria

## Abstract

**Background:**

There are indications that teachers have limited knowledge about attention deficit hyperactivity disorder (ADHD), despite its high prevalence in childhood and its long-term effects on students such as academic underachievement, reduced self-esteem, and social and behavioural difficulties. This study is therefore aimed at assessing the effect of an ADHD training program on the knowledge of ADHD among primary school teachers in Kaduna, Nigeria and their attitudes towards pupils with ADHD.

**Methods:**

This was a randomized controlled trial involving 84 primary school teachers in the intervention group and 75 teachers in the control group. Participants in the intervention group received an initial 3-h training with a one-and-a-half hour booster session 2 weeks later using the World Health Organisation MhGAP-IG module on behavioural disorders focusing on ADHD. Outcome measures were knowledge of ADHD, attitude towards ADHD, and knowledge of behavioural intervention.

**Results:**

Controlling for baseline scores, the intervention group had significantly higher post intervention scores on knowledge of ADHD, lower scores on attitude towards ADHD (i.e. less negative attitudes), and higher scores on knowledge of behavioural intervention compared with the control group respectively. The intervention showed moderate to large effect sizes. The booster training was associated with a further statistically significant increase in knowledge of ADHD only.

**Conclusions:**

The training program significantly improved the knowledge and attitudes of the teachers in the intervention group towards ADHD. Considerations should be given to incorporating ADHD training programs into teacher-training curricula in Nigeria, with regular reinforcement through in-service training.

## Background

Attention deficit hyperactivity disorder (ADHD) is one of the common childhood neuro-developmental disorders which is often associated with disturbed classroom behaviour [1] and one of the most frequent reasons for referral to school psychologists [2]. The inattention, impulsivity and hyperactivity which are the symptoms of ADHD are usually evident in the classroom, placing teachers in a unique position to identify and refer such students for further assessment [3]. Despite this, studies have found that teachers have limited and inaccurate knowledge about ADHD and often provide inappropriate information about the condition to parents [4].

Findings from previous studies in Nigeria and other developing countries [5–10] indicate that teachers have limited knowledge of ADHD. For example, Jimoh [11] studied 250 teachers from 10 public and 10 private schools in Lagos, Nigeria and reported deficiencies in their knowledge as well as negative attitudes towards pupils with ADHD. Similarly, Adeosun et al. [10] reported negative attitudes towards pupils with ADHD among 144 primary school teachers in Lagos. Not only in Nigeria and other developing countries such as Trinidad and Tobago [12] but even in developed countries such as the UK [13], teachers' attitudes towards ADHD and the role of pharmacological treatment remains unfavorable. The role of teachers becomes even more important in developing countries because parents may not have access to other supports and information sources to help them support their children with ADHD.

As children spend the majority of their time in schools [14] and interact with teachers in a variety of ways on a daily basis [15], practitioners rely on teachers to provide information to assist in establishing the diagnosis of ADHD. Carey [16] found that more than half of the 401 paediatricians studied relied solely on information from school reports to diagnose ADHD.

Furthermore, teachers are essential in the implementation, support and evaluation of recommended treatment plan for children with ADHD [17]. Also, teachers make recommendations, appropriate or inappropriate, about ADHD to the parents, who tend to follow such recommendations [16, 23]. In turn, parents frequently turn to teachers for information about ADHD [19]. Di Battista and Sheperd [20] found that teachers provided incorrect and unsuitable advice to parents of children with ADHD which many of them followed. Thus, the knowledge that teachers have about ADHD affects their behaviour and attitudes towards affected children. For example, a literature review of North-American studies by Sherman et al. [21] suggests that teacher factors such as their view on treatment options, and types of strategies used in the classroom can have huge influence on the educational outcome of children with ADHD. Also teachers with limited knowledge of ADHD may fail to identify children with symptoms who may otherwise benefit from assessment and treatment [17]. Negative teachers’ attitude may result in demotivation and self-deprecation by students affected by ADHD [22]. A recent cross-national comparisons of teachers’ knowledge and misconceptions of ADHD involving nine countries including South Africa [23] emphasised the importance of greater teachers’ knowledge of ADHD in many aspects including in promoting help-seeking. Therefore, in view of the importance of improving teacher’s knowledge and attitude towards ADHD, the current study was designed to assess the effect of an ADHD training program on the knowledge and attitudes of primary school teachers in Kaduna, Nigeria. To our knowledge, this is the first study to specifically evaluate the effect of training teachers on ADHD in Nigeria.

## Methods

This was a randomized controlled trial with intervention and waitlist control groups. The target group was teachers in public and private primary schools in Kaduna, North West Nigeria. Kaduna is one of the most cosmopolitan cities in Nigeria with sizeable proportions of every major ethnic group.

Nigerian public schools are government-run schools predominantly attended by students from families with lower income [24] and face challenges of operational quality, absence of required facilities, lack of parental commitment to school activities and high rate of bullying [24]. In contrast, private schools in Nigeria are owned by individuals, attended by families with higher income and foster a greater sense of community and are more responsive to parents and students [24].

At the time of the study, the population of teachers in government and private primary schools in Kaduna metropolis was 36,492 and 19,283 respectively in the private schools [25].

### Sample size determination

The sample size for the study was calculated using the formula for comparing two means [26]:

$${\text{n}} = 2 {\text{F}}\left( {\sigma /{\text{d}}} \right)^{ 2}$$

where n = the sample for each of the intervention and control groups, F = 7.85 is a factor which is based on power of 80 and 0.05% level of significance [20], σ = the standard deviation for the outcome measure, d = the difference we hypothesise will be found between the treatment and control groups. We are assuming that the training will result in the treatment group having a half standard deviation (0.5) better knowledge of the intervention content than the control group hence; the sample size will be

$${\text{n}} = 2 {\text{F}}\left( {\sigma /{\text{d}}} \right)^{ 2} ,$$

$${\text{n}} = 2 \times 7. 8 5\left( { 1/0. 5} \right)^{ 2} ,$$

$${\text{n}} = 6 2. 8 \approx 6 3.$$

Thus, a sample of 63 teachers in each of the intervention and control groups was identified as adequate to identify a post intervention difference of half a standard deviation in teachers’ knowledge based on 80% power and 0.05% level of significance.

In order to compensate for possible non-response, the final target sample size was increased to 70 teachers in each group. However, due to an agreement with headmasters to select only one of two teachers from each class (so as not to leave any class unattended during the training) the teachers that eventually participated were 84 in the intervention group and 75 in the control group. The teachers selected in this procedure exceeded the sample size but all were accommodated in the training to avoid leaving some disappointed.

### Sampling and study procedure

The teachers in the intervention group were selected from primary schools in a local government area different from that of the control group in order to avoid contamination. The 23 local government areas in Kaduna metropolis were listed in alphabetical order, and two local government areas (Kaduna South and Chikun) were randomly selected. The inclusion of all the 23 local government areas, with half of the regions being in the intervention group and the other half being in the control group, would have been ideal but this was logistically difficult within the resources available for this study. Chikun was randomly assigned to control group and Kaduna South to intervention group by balloting. Next, schools in the two local government areas were stratified into public schools and private schools. The schools in each group were listed in alphabetical order and assigned numbers. This was then followed by selection of schools from each group using table of random numbers. Headmasters of the intervention schools were asked to identify teachers in the schools who would like to be trained on ADHD.

In order to have at least one teacher to manage each classroom during the training, the headmaster used balloting to select one teacher if both teachers in the same classroom indicated interest in participating in the training. The teachers selected in this procedure exceeded the sample size but were accommodated in the training to avoid leaving some disappointed. Similarly, the head teachers of the control group schools also selected teachers who indicated interest in ADHD training in the future. Similar balloting technique was used to select eligible teachers until the sample size was reached. For logistical reasons, teachers were trained in their own schools using either a big classroom or the library. A total of seven schools participated in the study: four schools in the control group and three schools in the intervention group. There were two public and one private school in the intervention group and two public and two private schools in the control group. The number of private and public schools selected was based on probability-proportional-to size (PPS) calculation using the teacher population as the basis. The training lasted for 3 h with a break of 10 min after each hour. The materials were reinforced with a second booster session of one-and-a-half hours 2 weeks later. The intervention and control groups completed the outcome measures at baseline and 1 week after the first 3-h training for the intervention group. The measures were repeated for the intervention group alone 1 week after the booster session.

### Measures

A sociodemographic questionnaire obtained information about the teachers’ characteristics such as age, gender, previous training on ADHD, teaching experience and qualifications.

The 27-item section B of the Self-report ADHD questionnaire (SRAQ) [27] was used to assess teachers’ knowledge of symptoms, diagnosis, treatment, nature, causes, and outcome of ADHD. Each item is answered as “True,” “False,” or “Don’t Know”. The SRAQ was derived from Knowledge of Attention Deficit Disorders Scale (KADDS) [28] and has acceptable internal reliability (α = 0.78 for the knowledge scale). The correct answers were summed into a knowledge score where higher scores indicate better knowledge of ADHD (range 0–27).

The ADHD Attitude Scale (section D) of the SRAQ [4] was used to assess teachers’ beliefs and attitudes about ADHD. It has 30-items scored on a 5-point Likert-type scale (1 = strongly disagree to 5 = strongly agree). Some items in the scale measured cognitive attitude (e.g. “*ADHD is an excuse for children to misbehave*”), others measured affective attitude (e.g. “*I would feel frustrated having to teach a child with ADHD*”), and some items tapped into behavioural component of attitude (e.g. “*Children with ADHD should not be taught in the regular school system like ours*”). The answers were summed to create an ADHD Attitude Scale where higher scores indicate more negative attitude (range 30–150, α = 0.79).

The knowledge of Behavioural Interventions Questionnaire (KBIQ) was used to assess the teachers’ knowledge of common classroom strategies for ADHD. The KBIQ was a 12-item instrument designed by the second author for the purpose of this study. Face validity for the KBIQ was established through peer review. Piloting among 15 teachers in a school not involved in the study confirmed clarity. Examples of items in the scale include:

“*The position where a child with ADHD sits in the classroom does not really affect their behaviour or learning as long as they feel comfortable”. “Children with ADHD may need extra breaks if a classroom activity requires lengthy periods of sitting”. “Punishing children with ADHD for bad behaviour is more effective in changing their behaviour than rewarding them for good behaviour”. “Frequent praise for a child with ADHD is not good for them as they become “big-headed” and start behaving badly*”.

Correct responses were scored as 1 while incorrect responses and don’t know were scored as 0. The correct answers were summed to create a KBIQ score where higher scores indicate better knowledge of behavioural interventions (range 0–12). The KBIQ showed good internal consistency (α = 0.82).

### The intervention

The intervention was taken from the World Health Organisation’s Mental Health Gap Action Programme Intervention Guide (MhGAP-IG) [29] which was developed to support the delivery of mental health interventions in non-specialist settings. The behavioural disorders module of the MhGAP covers ADHD. We used the content for the training of primary school teachers regarding ADHD. The module covers the symptoms of ADHD, associated impairment, other conditions that need to be excluded, and the treatment options including behavioural interventions and medication. The participants were also trained on classroom management strategies for children with ADHD. The training was delivered by the first author using PowerPoint presentations, clinical vignettes, role plays, small group discussions and videos. The intervention was offered to the waitlist control group when it became evident that it was helpful for the intervention group. We confirmed that the control group did not receive any similar intervention before the last outcome measures were collected.

### Data analysis

The data was analysed with SPSS version 16. Chi-square test and independent sample *t* test were used to assess differences between the intervention and control groups. Analysis of co-variance (ANCOVA) was performed on the three outcome measures to determine the effect of the intervention. The post intervention scores were used as the dependent variables while the fixed factor was the treatment group. Pre-intervention scores were entered as covariates and controlled for. Age was also controlled for in the ANCOVA for knowledge of ADHD because age correlated significantly with this outcome variable with older teachers having less knowledge (r = −0.2, p = 0.05). Similarly, gender was entered as an additional fixed factor in the ANCOVA for Attitude towards ADHD because males had significantly more negative attitudes than females {(M = 97.81 SD = 9.74) vs (M = 92.67 SD = 9.07), t = 2.13, p = 0.03}. Cohen d effect sizes were calculated with 0.20–0.49, 0.50–0.79 and 0.8 or higher representing small, medium and large effect sizes respectively [30]. For the intervention group alone, paired sample *t* tests were used to compare the first post intervention scores on outcome measures and the post booster-session scores. Effect sizes were also calculated as above.

## Results

A total of 159 primary school teachers from four public and three private schools participated in this study (84 in the intervention group and 75 controls). There were two public and one private schools in the intervention group and two public and two private schools in the control group. The number of private and public schools selected was based on PPS calculation using the teacher population as the basis. In the intervention group, 84 teachers completed the baseline measures and attended the first training session, 76 teachers completed the first post intervention measures 1 week later. Seventy-six teachers attended the booster session but 75 completed the post booster measures 1 week after. In the control group, 75 teachers filled the baseline measures while 71 teachers were available for the follow up measures which took place the same week as for the intervention group.

### Socio-demographic characteristics of participants

The mean age of the teachers was 42.46 ± 8.03 years and with an average of 14.30 years (*SD* = 8.13 years) of teaching experience. Table 1 shows that teachers in the two groups were not statistically different in gender, type of school, qualifications, classes currently taught, having additional training on ADHD, ever teaching pupils with ADHD, number of ADHD workshops previously attended, number of ADHD articles read, whether previous education involved training on ADHD and whether their schools employed people specifically to help pupils with ADHD. However, teachers in the intervention group were significantly older, had more years of teaching experience, and smaller classes, while the teachers in the control group were more likely to have ever requested for ADHD evaluation for their pupils as well as taught more children with ADHD.

**Table 1:** Socio-demographic characteristics, teaching history, and past experience of ADHD between the treatment and control groups

Variables	Treatment group (n = 84)	Control group (n = 75)	*t* test or χ^2^	p
Age, mean (SD)	44.81 (9.64)	39.83 (7.68)	4.10	<0.001*
Gender, n (%)				
Male	12 (75.0)	4 (25.0)	3.5	0.06
Female	72 (50.3)	71 (49.7)		
Type of school				
Public	58 (52.3)	53 (47.7)	0.05	0.082
Private	26 (54.2)	22 (45.8)		
Qualifications				
NCE	57 (52.8)	51 (47.2)	1.5	0.82
Degree	20 (50.0)	20 (50.0)		
PGD	2 (50.0)	2 (50.0)		
Grade 2	1 (100.0)	–		
Masters	4 (66.7)	2 (33.3)		
Class currently taught				
Nursery	10 (50.0)	10 (50.0)	1.0	0.60
Primary 1–3	36 (49.3)	37 (50.7)		
Primary 4–6	38 (57.6)	28 (42.4)		
Previous education involving ADHD				
Yes	17 (39.5)	26 (60.5)	3.16	0.08^Y^
No	65 (57.8)	49 (42.2)		
Additional training on ADHD				
Yes	14 (58.3)	10 (41.7)	0.3	0.56
No	70 (51.9)	65 (48.1)		
Ever taught pupil with ADHD				
Yes	48 (49.5)	47 (50.5)	1.0	0.31
No	38 (57.6)	28 (42.4)		
Ever requested ADHD evaluation				
Yes	5 (25.0)	15 (75.0)	5.9	<0.02^Y^
No	79 (56.8)	60 (43.2)		
Does your school employ helpers for pupils with ADHD				
Yes	4 (30.8)	6 (69.2)	2.8	0.10
No	80 (54.8)	66 (45.2)		
No of years teaching	15.62 (8.48)	12.83 (7.49)	2.19	0.03*
No of pupils in the class	30.68 (8.78)	44.44 (21.54)	−3.40	<0.001*
No of workshops attended on ADHD	0.14 (0.58)	0.19 (0.51)	−0.50	0.62
Hour of ADHD training had before	0.63 (1.40)	1.03 (2.09)	1.42	0.16
No of articles read on ADHD	0.48 (1.92)	0.41 (1.30)	0.24	0.81
No of students with ADHD ever taught in the past	2.90 (6.51)	6.43 (13.62)	−2.12	<0.04*

^*Y*^Yates correction

* Significant at p < 0.05

### Effectiveness of the intervention

At baseline, the scores on knowledge and attitude towards ADHD were not significantly different between the groups but the intervention group scored significantly higher on knowledge of behavioural intervention (Table 2). However, post-intervention, the intervention group scored significantly higher on Knowledge of ADHD (t = 5.270, df = 145, p = 0.0001), knowledge of behavioural interventions for ADHD (t = 3.594, df = 145, p = 0.005), and significantly less on negative attitude towards ADHD (t = −2.838, df = 145, p = 0.0001). As shown in Table 2, ANCOVA showed statistically significant differences in the post-intervention scores on all three outcomes between the two groups having controlled for the pre-intervention scores and other confounders. The intervention group scored significantly higher on knowledge of ADHD {F (1,143) = 38.1, p = 0.000}. The intervention explained 21% of the variance in the post intervention knowledge of ADHD scores with a large effect size of 0.9. Similarly, the training programme showed a statistically significant effect on attitude towards ADHD scores {F (1,143) = 11.0, p = 0.001} and explained 7.1% of the variance with a moderate Cohen’s effect size (d) of 0.5. Finally, a statistically significant treatment effect on knowledge of behavioural intervention {F (1,143) = 9.5, p = 0.002} was observed with a moderate Cohen’s effect size (d) of 0.6.

**Table 2:** Comparisons between intervention group and control group on outcome measures (knowledge of ADHD, attitude to ADHD, and knowledge of behavioural intervention)

Variable	Intervention group mean (SD)			Control group mean (SD)			F value (1,143)	p value	Effect size (Cohen d)
	Pre n = 76	Post n = 76	Difference	Pre n = 71	Post n = 71	Difference			
Knowledge of ADHD	11.03 (4.13)	14.74 (3.25)	t = −8.33 p < 0.001	11.04 (4.01)	11.80 (3.50)	t = −1.67 p = 0.10	38.1	0.000	0.9
Attitude towards ADHD	93.59 (10.28)	88.08 (7.67)	t = 5.22 p < 0.001	93.49 (8.14)	92.37 (8.94)	t = 0.93 p = 0.35	11.0	0.001	0.5
Knowledge of behavioural intervention	7.39 (2.88)	8.37 (2.12)	t = −3.11 p = 0.003	6.54 (2.69)	7.04 (2.36)	t = −1.42 p = 0.16	9.5	0.002	0.6

### Impact of booster session

Table 3 shows paired *t* tests which indicate that the second booster training was associated with a statistically significant further increase in knowledge of ADHD but no further increase in knowledge of behavioural intervention or a further reduction in negative attitude towards ADHD.

**Table 3:** Intervention group only: within group differences in post intervention and post booster scores on outcome measures (knowledge of ADHD, attitude to ADHD, and knowledge of behavioural intervention)

Continuous variables	Post-intervention n = 75	Post-booster n = 75	t	df	p
Knowledge of ADHD score	14.83 (±3.18)	15.48 (±3.53)	−2.12	74	0.04*
Attitude to ADHD score	88.13 (±7.71)	86.92 (±8.95)	1.22	74	0.23
Knowledge of behavioural intervention	8.40 (±2.11)	8.81 (±2.07)	−1.67	74	0.10

*Significant at p < 0.05

## Discussion

This is a randomized controlled trial of the effect of ADHD training on the knowledge and attitude of primary school teachers in Kaduna, North West Nigeria towards this condition. Teachers in the intervention group were trained using a standard ADHD training program for 3 h in the first session and one-and-a-half hours in the second booster session 2 weeks later. Compared with the control group, the ADHD training program demonstrated a statistically significant increase in knowledge of ADHD and its behavioural management, and improved attitude towards affected children.

The need for this type of study in Nigeria is evidenced by extant literature indicating low levels of knowledge of ADHD and negative attitude towards affected children by Nigerian teachers. Further support for the need for this intervention comes from the current study which showed the teachers had limited exposure to ADHD training. For example, only a third of the teachers reported that their previous training included ADHD. Also less than a fifth of the participants had had additional training on ADHD in spite of an average of 14 years of teaching experience. These observations become more pertinent when it is considered that the 5% prevalence of ADHD means that every classroom is likely to have one or more children with the condition [18, 31].

The improvement in knowledge of ADHD, attitude towards affected children, and knowledge of ADHD-related behavioural management following the intervention in this study is similar to findings from previous studies using a variety of training methods and platforms such as provision of written materials [9], one point training [32], short-term intervention (1 week) [33], as well as internet based training. These have all shown rapidly improved knowledge about ADHD, with benefits lasting for up to 6 months [34, 35].

The study by Sarraf et al. [9] is particularly relevant to areas with very limited resources. They conducted a two-method training on ADHD among 67 primary school teachers in Iran. The first method involved a 2-day workshop while the second method was a nonattendance education group. The latter group was given ADHD related booklets to study with the precise educational content similar to that of the workshop group. Post-test questionnaires were given to the workshop group after the 2 days of training. The nonattendance group who had studied the related booklets was assessed after 10 days. They found that both the nonattendance education method and workshop method were effective in promoting teachers’ knowledge of ADHD. However, the workshop education was more effective in changing attitude and improving knowledge of behaviour management of students with ADHD. This study suggests that where resources are insufficient to support face to face training, providing teachers with written information about ADHD could in the least improve their knowledge of the condition.

## Limitations of the study

Due to time and resource constraints, the duration of the intervention was short comprising of a 3-h session followed 2 weeks later by a one-and-a-half hour booster training. Also, the participants were randomised at school level rather than as individuals. The latter would have been ideal but would have been impractical within the resources available for the study. Masking was not feasible which means that socially desirable responding could have contributed to the better outcomes among the intervention group. The study used a waitlist control group (rather than an active control group) and treatment trials using waitlist controls tend to show better outcomes. The inclusion of all the 23 local government areas in the study area, with half of the regions being in the intervention group and the other half being in the control group, would have been ideal but this was logistically difficult within the resources available for this study. The administrative structure of the schools made it pragmatic for headmasters to be involved in identifying participants. However, this may have introduced bias compared with if teachers were recruited directly. Finally, the long term impact of the training is uncertain as we only have short term outcomes.

## Conclusion

ADHD is a prevalent neuro-developmental disorder affecting 3–7% of school-aged children. This suggests that every classroom of 25 children would have at least one child with ADHD. However, findings from previous studies indicate that teachers have low knowledge of ADHD as well as negative attitude towards affected children. This study showed that one session of ADHD training using a standard readily available training package can improve teacher’s knowledge and attitude towards ADHD. Thus consideration should be given to the integration of ADHD training programs into teacher training programs and inclusion of ADHD in the continuing professional development training of already qualified teachers in Nigeria.

## Abbreviations

ADHD: attention deficit hyperactivity disorder
mhGAP-IG: Mental Health Gap Action Programme—Intervention Guide
SD: standard deviation
SRAQ: self report ADHD questionnaire
KADDS: Knowledge of Attention Deficit Disorders Scale
KBIQ: Knowledge of Behavioural Intervention Questionnaire
WHO: World Health Organisation
ANCOVA: analysis of covariance
